# Fractional spinal anesthesia and systemic hemodynamics in frail elderly hip fracture patients

**DOI:** 10.12688/f1000research.130387.3

**Published:** 2023-07-31

**Authors:** Fredrik Olsen, Mathias Hård af Segerstad, Keti Dalla, Sven-Erik Ricksten, Bengt Nellgård

**Affiliations:** 1 Anesthesia and Critical Care, Sahlgrenska Academy, University of Gothenburg, Gothenburg, Sweden

**Keywords:** Spinal Anesthesia, hip fracture surgery, cardiac output, hypotension, elderly patients

## Abstract

**Background:** Systemic haemodynamic effects of intrathecal anaesthesia in an aging and frail population has not been well investigated. We examined the systemic haemodynamics of fractional spinal anaesthesia following intermittent microdosing of a local anaesthetic and an opioid.

**Methods: **We included 15 patients aged over 65 with significant comorbidities, planned for hip fracture repair. Patients received a spinal catheter and cardiac output monitoring using the LiDCOplus system. All measurements were performed prior to start of surgery. Invasive mean arterial pressure (MAP), cardiac index (CI), systemic vascular resistance index (SVRI), heart rate and stroke volume index (SVI) were registered. Two doses of bupivacaine 2.25 mg and fentanyl 15 µg were administered with 25-minute intervals. Hypotension was defined as a fall in MAP by >30% or a MAP <65 mmHg.

**Results:** The incidence of hypotension was 30%. Hypotensive patients (n=5) were treated with low doses of norepinephrine (0.01-0.12 µg/kg/min). MAP showed a maximum reduction of 17% at 10 minutes following the first dose. CI, systemic vascular resistance index and stroke volume index decreased by 10%, 6%, and 7%, respectively, while heart rate was unchanged over time. After the second dose, none of the systemic haemodynamic variables were affected.

**Conclusions:** Fractional spinal anaesthesia administered prior to surgery induced a minor to moderate fall in MAP, mainly caused by a reduction in cardiac output, induced by systemic venodilation, causing a fall in venous return. Our results are contrary to the widely held belief that hypotension is mainly the result of a reduction of systemic vascular resistance.

## Introduction

A hip fracture in a frail elderly patient poses a major challenge in anesthesiology as these patients are mostly presented off-hours and many have comorbidities. ^1^ The 30-day mortality among hip fracture patients is as high as 6-10%. ^2^ Many factors have been associated with mortality, including time to start-of-surgery, ^3^ cementation of hemi- or total arthroplasty, ^4^ male sex, ^5^ and preoperative morbidity assessed by the American Society of Anaesthesiologist (ASA) risk score and Nottingham Hip Fracture Score (NHFS). ^6^^,^^7^ Preoperative cardiology consulting, however, rarely affects surgical management, but could alter anesthesiologic management. ^8^

The peri-operative anaesthesia strategy for the management of the frail hip fracture patient differs worldwide: where many centers give general anaesthesia, while particularly in northern Europe, neuraxial anaesthesia is the preferred technique. ^9^ However, both techniques frequently induce hypotension, requiring fluid resuscitation and/or the need for vasopressors. ^10^ Perioperative hypotension is a problem predisposing patients to organ hypoperfusion with consequences such as myocardial injury, delirium and renal failure. ^10^^,^^11^ The physiological origin of the hypotension is unclear, but many anesthesiologists believe that the decrease of systemic vascular resistance (SVR) is the main cause of hypotension, ^12^ while others believe that hypotension is caused by a fall in cardiac output ^13^ or a combination of both.

In our hospital, we routinely administer neuraxial anaesthesia for hip fracture surgery. We have a vast experience with this technique and in the present study we utilized the continuous spinal anaesthesia (CSA) technique to elucidate the hemodynamic response to fractional dosing without the influence of surgical manipulation. The use of CSA is well recognized to have a limited effect on hypotension. ^14^ It also allows us to study the hemodynamics in a prolonged period prior to surgery, minimizing the potential for other factors such as positioning, sedation and surgical stress to influence the measurements in a way a single shot spinal does not. The hemodynamics were monitored with LiDCOplus. LiDCOplus is a validated system in which a lithium dilution technique is used to calibrate the arterial pulse contour analysis. ^15^ The LiDCO system has previously been used in hip fracture patients in the perioperative setting. ^16^^–^^18^ The advantage of LiDCO compared to other invasive hemodynamic devices is that lithium can be injected in a peripheral venous cannula and then lithium concentration is captured through a standard 20G arterial cannula. Thus, we could avoid more invasive monitoring using central venous catheters, femoral arterial cannula, or the Swan-Ganz catheter.

The aim of the present study was to investigate the systemic haemodynamic response to fractional spinal anaesthesia, in a group of elderly and comorbid patients with hip fracture, using the LiDCOplus system to monitor pre-surgical haemodynamic changes over time.

## Methods

Ethical approval was granted by the Gothenburg Regional Ethical Review Board (Dnr 2020-05684). During the study period we screened daily for patients planned for hip fracture surgery and these were identified through the theatre planning software (Orbit, TietoEVRY, Espoo, Finland). Inclusion criteria were: 1) patient with hip fracture, 2) >65 years of age, 3) ASA ≥2, 4) scheduled for neuraxial anaesthesia and 5) mentally fit to give written informed consent or permission by next-of-kin in cognitive impaired patients. Exclusion criteria were: a) lithium or anticoagulation medication, b) planned for general anaesthesia, c) ongoing atrial fibrillation, d) if surgery was delayed >72 hours, e) lack of informed consent and f) patient agitation requiring intermittent sedation. The study was carried out in accordance with the Declaration of Helsinki (2000). Finally, inclusion rate was dependent and affected by the primary investigator’s availability and the operative capacity, by recruiting consecutive cases within these limitations we aspired to recruit a representative selection of patients with regards to self-reported gender and level of comorbidity. NHFS was calculated, the scale going from 1-10 with higher numbers correlated to a higher 30-day mortality. ^6^^,^^19^ ASA grade along with defined laboratory values, demographic data and chronic disease were also recorded after study inclusion.

After arriving to the preoperative area, patients were given 5 liters of oxygen on a face mask and ECG and pulse-oximetry monitoring was started. Oral premedication with standardized doses of paracetamol (1 g) and oxycodone (5 mg) was given orally, followed by the placement of a venous 18G cannula in an antecubital vein and a radial arterial catheter (20G). The patient was also given a fascia iliaca compartment (FIC) block, or an ultrasound guided femoral nerve block with ropivacaine 3.5 mg/mL 20-40mL, to minimize discomfort and to avoid sedation or systematic analgesia when positioning for the neuraxial block. In addition, the LiDCOplus (LiDCO Group Plc, London, England) system was set up and calibrated according to manufacturer’s instructions. Calibration was performed with 0.3-0.45 mmol lithium chloride injection based on body weight. After calibration and baseline parameter registration, the LiDCOplus system provided cardiac output variables and based on these and the invasive blood pressure, haemodynamic variables could be derived.

Following aseptic skin preparation of the lumbar area, a subarachnoid puncture by a 18G Tuohy needle was performed either between the L2 - L3 or the L3 - L4 interspaces, preferably using a mid-line approach. An intrathecal catheter 20G was then inserted 4-5 cm into the intrathecal space. This technique of a continuous spinal anaesthesia (CSA) was performed on all patients by a dedicated physician (FO).

An intrathecal mixture (10 mL) containing 1.5 mg/mL bupivacaine and 10 μg/mL fentanyl was prepared. Intrathecal anaesthesia was induced by giving 1.5 mL (2.25 mg of bupivacaine and 15 μg of fentanyl) of the mixture, followed by a second 1.5 mL injection after 25 min ( *i.e.*, a total intrathecal dos of 4.5 mg of bupivacaine and 30 μg of fentanyl). Sensory level was monitored by “cold spray”. Hemodynamic recordings were documented every five minutes up until 45 minutes after initial intrathecal dose when research monitoring was also terminated. The patient was then operated upon in the pre-planned time slot and was further managed at the discretion of the attending anesthetist.

Mean arterial blood pressure (MAP) was maintained, when needed, with a norepinephrine infusion to target a MAP >65 mmHg or to avoid more than 30% decline in MAP from baseline. In addition to SaO _2_ and ECG, the following parameters were recorded: cardiac index (CI), stroke volume index (SVI), systemic vascular resistance index (SVRI), systolic arterial pressure, (SAP), diastolic arterial pressure (DAP) and nor-epinephrine doses over time. Finally, effective arterial elastance (EA) was calculated by the formula; 0.9×SAP/SV. ^20^ For indexing parameters, the Du Bois and Du Bois formula for body surface area (BSA) was used. ^21^

### Statistics

Statistical analysis was performed with RStudio for Mac (version 1.2.5033) and GPower version 3.1.9.6 (Franz Faul, Universität Kiel, Germany) to determine sample size. Normality was assessed with Shapiro-Wilk test prior to deciding appropriate variation testing, one-way repeated measures ANOVA for normally distributed and Friedmann test for non-normal distributions. For repeated measures, ANOVA was utilized to study changes in haemodynamic variables over time. A p-value <0.05 was considered statistically significant. A sample size of n=13 patients for the repeated measured ANOVA was needed to have an 80% power (β=0.20) for detection the effect size F=0.25 (α=0.05).

## Results

**Figure 1.**
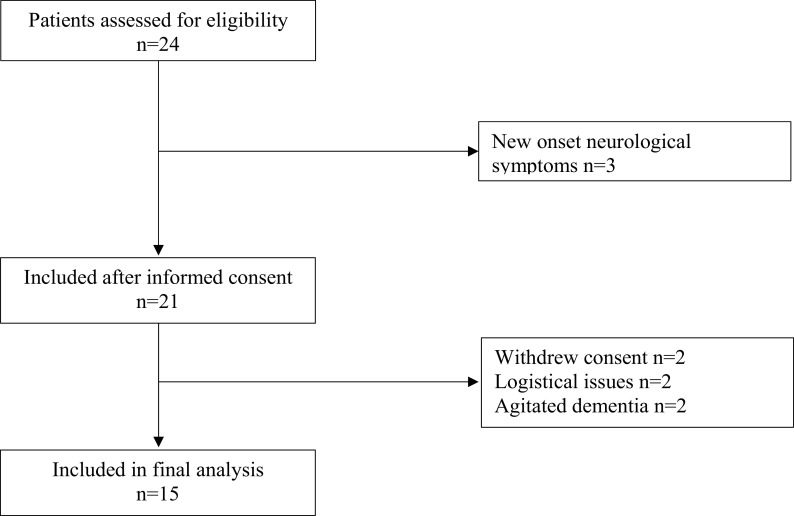
Consort diagram.

The clinical trial profile is shown in Figure 1. A total of 24 patients were eligible for the study inclusion, of whom 15 were finally included. Two patients withdrew consent, two patients were excluded due to logistical issues, two patients were excluded for agitated dementia and finally, three patients were excluded due to having new onset of neurological symptoms. Hypertension was the dominant comorbidity present in 73% of patients, while dementia was found in 47%. Prior or present malignancy was found in 33% of patients. Further demographic data of the studied patients are summarized in Table 1. The study population had a median age of 89 years and consisted primarily of women (12/15). The median ASA grade was 3 (range 2-4) and the median NHFS score was 5 (range 4-7). None of the patients had significant arrythmias during the experimental procedure and none required vasopressor support prior to the first intrathecal dose of the bupivacaine/fentanyl mixture was given.

**Table 1.** Demographic data.

	Overall (N=15)
**Gender**, n (%)	
Female	12 (80%)
Male	3 (20%)
**Age**, (y; range)	89 (69-94)
**ASA**, n (%)	
2	4 (27%)
3	10 (67%)
4	1 (7%)
**NHFS**, n (%)	
4	6 (40%)
5	3 (20%)
6	4 (27%)
7	2 (13%)
**Body mass index** (kg/m ^2^)	22.0 (3.71)
**Body surface area** (m ^2^)	1.64 (0.133)
**Serum haemoglobin** (g/L)	113 (20.4)
**Previous myocardial infarction** n (%)	2 (13%)
**Hypertension** n (%)	11 (73%)
**COPD** n (%)	5 (33%)
**Dementia** n (%)	7 (47%)
**Malignancy** n (%)	5 (33%)

ASA: American Society of Anesthesiologists risk score, NHFS: Nottingham Hip Fracture Score, COPD; chronic obstructive pulmonary disease. Values are mean±SD, Age is median (range).

Sensory and motor functions were assessed, revealing that all patients had satisfactory levels of sensory block at minimum (>Th 12) documented with sensation to cold or painful reaction to flexion of the hip, which is the same assessment used for the single shot spinal prior to surgery. In mentally intact patients, all had a temperature discrimination demonstrated by a sensory block <Th 8 level. Further, we noticed a high incidence of retained motor function after the initial neuraxial 1.5 mL dose (2.25 mg bupivacaine and 15 μg fentanyl), possibly due to a less dense blockade.

**Figure 2.**
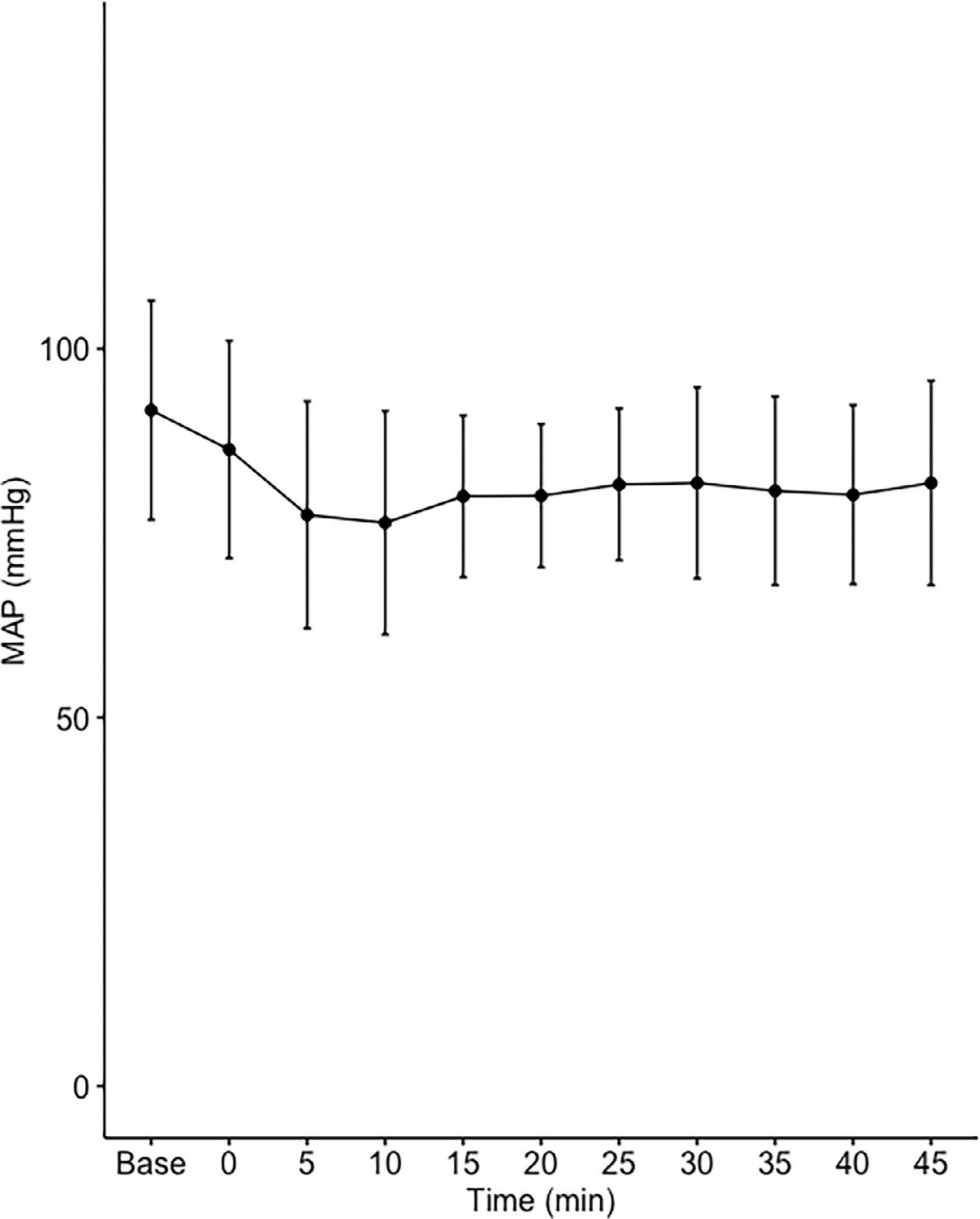
Changes in mean arterial pressure over time (mean±1SD).

**Figure 3.**
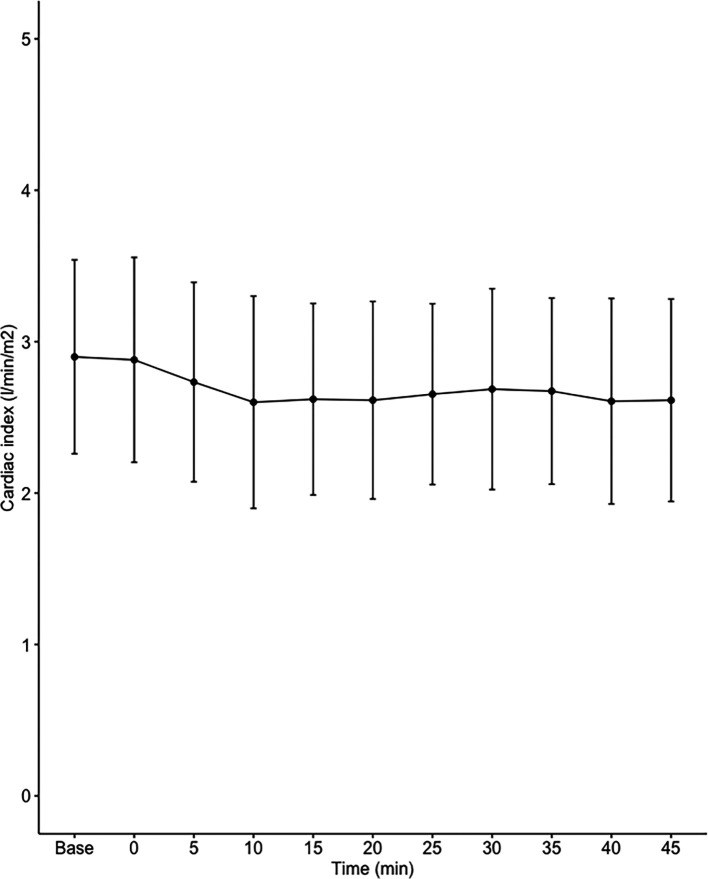
Changes in cardiac index over time (mean±1SD).

**Figure 4.**
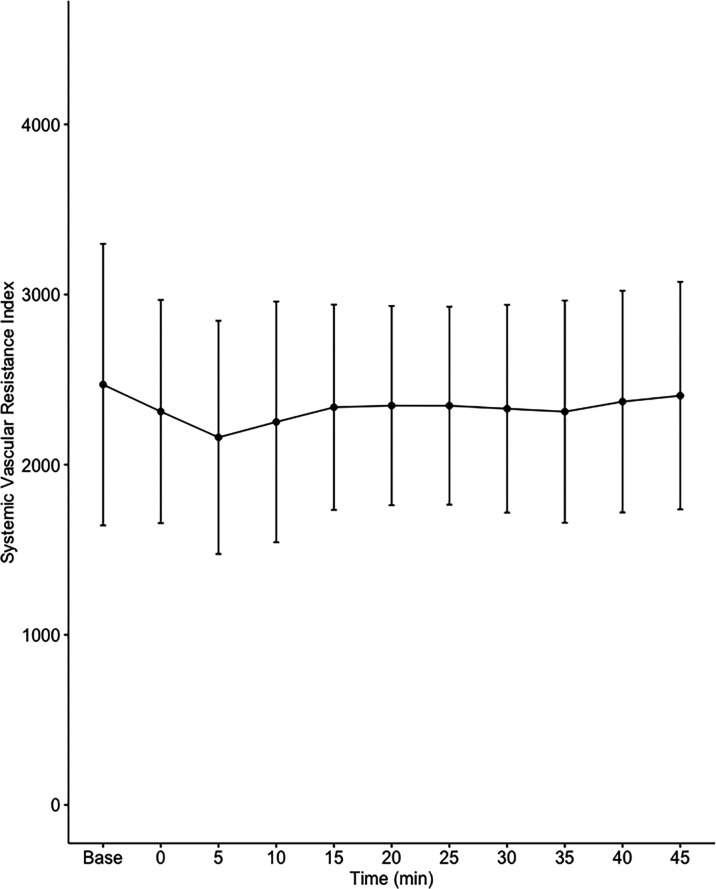
Changes in systemic vascular resistance index over time (mean±1SD).

**Figure 5.**
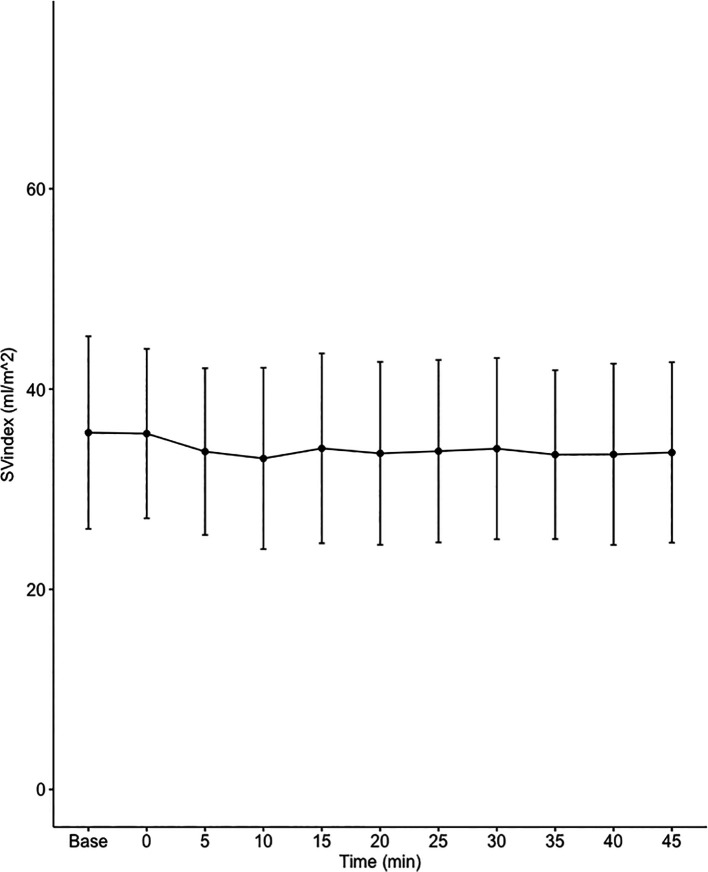
Changes in stroke volume index (SVI) over time (mean±1SD).

**Figure 6.**
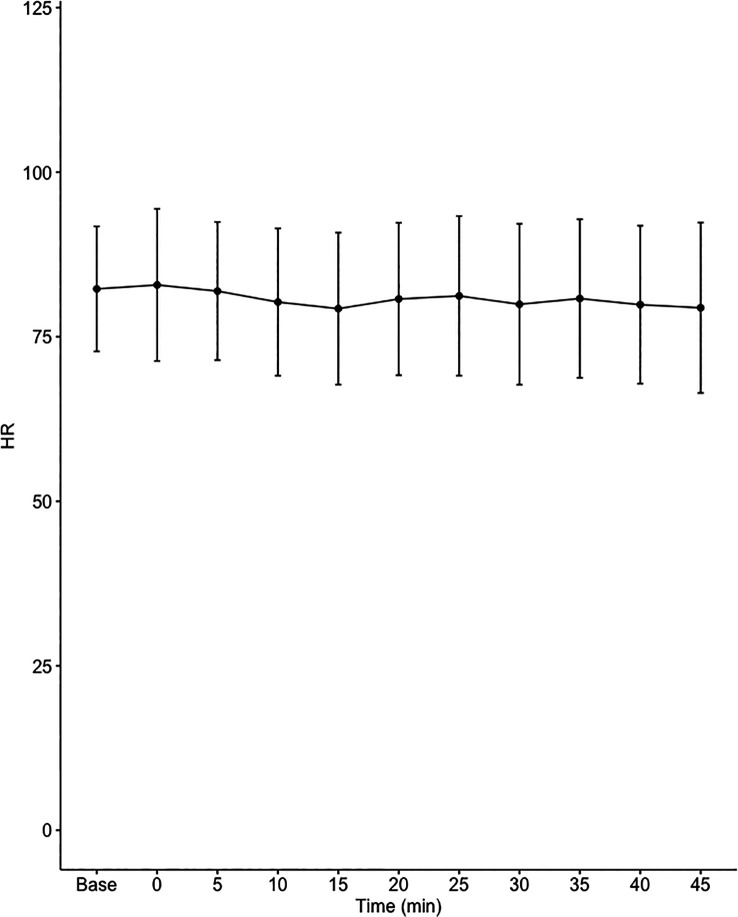
Changes in heart rate (HR) over time (mean±1SD).

**Figure 7.**
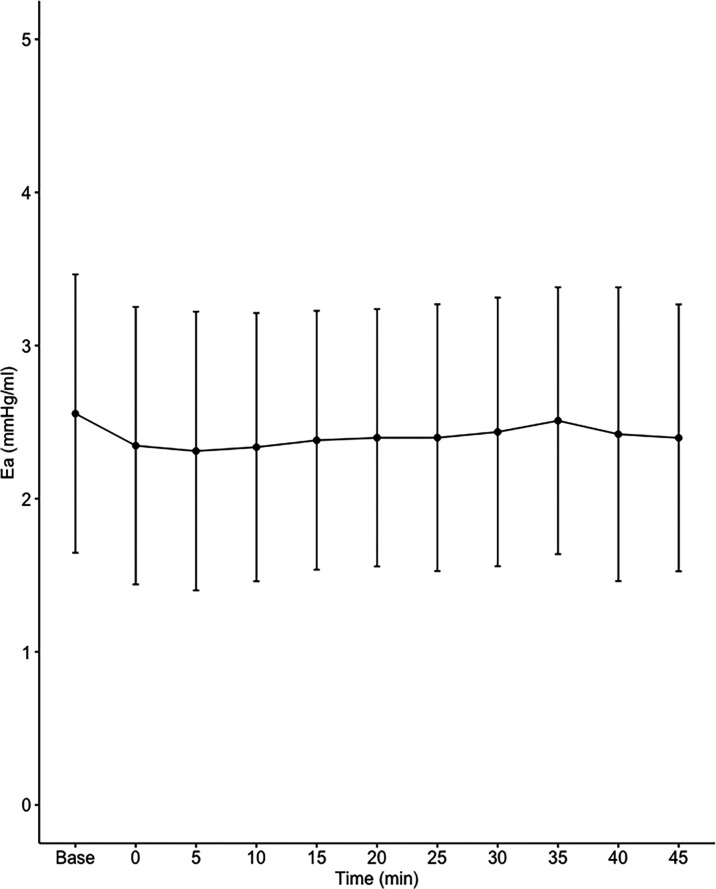
Changes in effective arterial elastance over time (mean±1SD).

Data on systemic haemodynamics are shown in Figures 2– 7. MAP, CI, SVRI, SVI and arterial elastance were all found to have normal distribution at each point of measurement according to Shapiro-Wilk’s test. After applying the one-way repeated measures ANOVA test, MAP, SVRI, SVI and CI all showed significant variance over time. Thus, MAP decreased by 17% from baseline with the lowest mean noted at 10 min after the first intrathecal dose was given. CI was reduced by 10% also after 10 minutes. SVRI showed a 6% reduction from baseline found directly after the intrathecal dose was given. SVI dropped by 7% with a lowest mean value at 10 minutes after anaesthesia induction and, finally, heart rate decreased non-significantly by 3% from baseline. Elastance did not show significant variation over time as measured by ANOVA. The largest reduction from the baseline value was -10% at 5 min after the initial spinal dose. One third (5/15) of the patients required norepinephrine infusion either to maintain a MAP>65 mmHg or to avoid a decrease by >30% from the baseline. The largest dose used was 0.12 μg/kg/min ( Table 2).

**Table 2.** Changes in hemodynamic variables over time.

	Baseline	0 min Dose 1	5 min	10 min	15 min	20 min	25 min Dose 2	30 min	35 min	40 min	45 min	ANOVA p value
**MAP (mmHg)**	92±15	86±15	78±15	76±15	80±11	80±10	82±10	82±13	81±13	80±12	82±14	0.00019
**CI (l/min/m ^2^)**	3.0±0.7	2.9±0.7	2.9±0.7	2.8±0.7	2.8±0.7	2.7±0.7	2.8±0.6	2.8±0.7	2.8±0.7	2.7±0.7	2.8±0.7	0.00015
SVRI (dynes*s/cm ^5^/m ^2^)	2470±827	2310±656	2160±685	2250±708	2340±603	2350±586	2350±582	2330±611	2310±653	2370±651	2410±669	0.017
**SVI (mL/m ^2^)**	35.7±9.6	35.6±8.5	33.8±8.3	33.1±9.1	34.1±9.5	33.6±9.1	33.8±9.1	34±9.1	33.5±8.4	33.5±9.1	33.7±9	0.021
**HR (1/min)**	82.3±9.5	82.9±11.6	81.9±10.5	80.3±11.2	79.3±11.5	80.7±11.6	81.2±12.1	79.9±12.2	80.8±12	79.9±12	79.4±12.9	0.472
**Ea (mmHg/mL)**	2.56±0.91	2.35±0.91	2.31±0.91	2.34±0.88	2.38±0.85	2.40±0.84	2.40±0.87	2.44±0.88	2.51±0.87	2.42±0.96	2.40±0.87	0.086
**Norepinephrine dose (μg/kg/min)**	0±0	0.003±0.01	0.01±0.02	0.02±0.03	0.02±0.03	0.02±0.03	0.02±0.03	0.02±0.03	0.02±0.03	0.02±0.03	0.02±0.03	0.002

MAP: mean arterial pressure, CI: cardiac index, SVRI: systemic vascular resistance index, SVI: stroke volume index, HR: heart rate, Ea: arterial elastance.

## Discussion

The main findings of the present study were that the hemodynamic aberrations after induction of fractional spinal anesthesia were minor to moderate, with a maximal fall in CI and MAP of 10-17%, 10 minutes following the first dose. After the second dose, no further changes in hemodynamics were seen arguing for a hemodynamic stability using CSA.

Interestingly, the maximal fall in SVRI was 6% and appeared early after the first dose with no further fall after the second dose. Thus, the MAP reduction was less than expected and the major contributor to the fall in MAP was a fall in CI, which explained almost 60% of the MAP reduction. This leads us to the conclusion that fractional spinal anaesthesia, as described in the present study, induces a vasodilation more prominent in systemic venous capacitance vessels, which decreases venous return and cardiac preload, as reflected by a decrease in SVI.

In this study, we defined hypotension as having a MAP <65 mmHg or a decrease of MAP >30% from the baseline level, a definition previously used in other studies. ^22^^–^^24^ Using this definition, the incidence of hypotension following single-shot spinal anaesthesia, has previously been described as being 28-69%, which is considerably higher than noted in the present study. ^25^^,^^26^ Hypotension from a spinal anaesthesia has been described by Butterworth ^27^ as a decrease in systemic vascular resistance and central venous pressure as a result of the sympathetic block, with vasodilation of both systemic resistance vessels as well as venous capacitance vessels, the latter causing redistribution of central blood volume to the lower extremities and splanchnic beds and thus impaired venous return. In the present study, MAP decreased by 17%, CI by 10%, SVRI by 6% and SVI by 7% at 10 minutes after the initial intrathecal injection. Our data imply that the MAP reduction can not be explained by a reduction in SVRI alone. The proportionally larger fall in CI implicates vasodilation more on the venous capacitance vessels, leading to reduced venous return and subsequently a fall in CI and MAP. These findings are in line with the findings of Jakobsson *et al.*, showing that a single shot of spinal anaesthesia (15 mg bupivacaine) induced hypotension in 50% of the patients, mainly caused by a fall in CI (20%) and SVI (15%). ^13^ Nakasuji *et al.*, on the other hand, found that the hypotension seen after a single-shot spinal anaesthesia (10 mg bupivacaine), in elderly patients, was mainly caused by systemic vasodilation and a fall in SVRI. ^12^ Our data exhibited a significantly smaller fall in SVRI than described by Salinas *et al.,* ^28^ possibly due to lower dosing achieved with intermittent dosing and subsequently lower levels of sympathetic block. Effective arterial elastance (Ea) incorporates all elements of total LV afterload, including vascular resistance, arterial compliance and characteristic impedance. ^29^ The finding that Ea was not affected by fractional spinal anaesthesia also indicates that the driving force of hypotension in the present study was systemic venodilation.

Single-shot neuraxial anaesthesia is predominantly used around the world in in this population of patients. Dosages have decreased over time and in our clinical routine, we rarely administer more than 2.5 mL of mixtures of local anesthetics and opioids. The injection time the mixture is given may have an effect on the hypotension severity and we await studies addressing this topic. In an interesting study by Szucs *et al.* ^30^ they used the “up-and-down” method described by Dixon and Massey in 1969 to find the lowest intrathecal dose of local anesthetic to provide adequate anaesthesia for a hip fracture operation. ^31^ They concluded that 0.24 mL of 5 mg/mL isobaric bupivacaine was enough as a single dose but still recommended a dose of 0.4 mL or more *i.e.* 2 mg. A more recent meta-analysis concluded that 6.5 mg of bupivacaine seems sufficient for hemodynamic stability, patient comfort and adequate motor block. ^32^ This is in concert with the present investigation where we gave dosages of 2.25 mg of isobaric bupivacaine although diluted to 1.5 mg/mL with sodium chloride and fentanyl. The volume in this study was larger, being 1.5 mL of the above-stated-mixture. However, most clinicians would not consider administering such low doses with the eminent risk of blockade failure and forcing the attending anaesthesiologist to give general anaesthesia, an alternative considered worse at the preoperative evaluation.

An attractive alternative to single-shot spinal is the continuous spinal anaesthesia (CSA) a technique described in the 1940’s ^33^ and improved by catheter insertion. ^34^ CSA has been associated with fewer incidents of hypotension *per se* and less severe episodes of hypotension. ^35^ This led us to revisit the technique at our clinic, as we have many hip fracture patients and many of them with variable severity of aortic stenosis. ^36^^–^^38^ We confirm the result of Minville *et al.* that by carefully giving a CSA we can avoid severe hypotensive incidents even in frail patients. ^35^ A side effect of this lower dosing was a less prominent motor blockage, also noted in the present investigation.

LiDCOplus is a semi-invasive, (needing an arterial catheter), validated method enabling us to register haemodynamic variables without the need for central venous- and/or Swan-Ganz catheters. The LiDCO system is therefore less invasive and does not require a higher degree of invasiveness than is routinely included in the normal clinical management of hip fracture patients at our hospital. To our knowledge, the golden standard technique for measuring cardiac output is the use of a Swan-Ganz catheter with thermodilution, but this technique is hardly doable in a cohort of frail elderly hip fracture patients. Calibrated cardiac output (CO) using LiDCO monitoring is relatively easy and quick to start in patients already in need of arterial cannulation and provides a deeper insight into perioperative haemodynamic changes.

### Limitation and strengths

With only 15 included patients the generalizability of our findings may be limited. We were restricted by the dynamic nature of running an effective emergency operating list, but also by the prevalence of patient anticoagulation therapy limiting the use of neuraxial anaesthesia in general and indwelling spinal catheter in particular. Haemodynamic monitoring with LiDCOplus gives us estimations of cardiac output after calibration where all other haemodynamic variables are derived from CO from the pulse power analysis and invasive blood pressure together with the heart rate. A strength of our study is that the cohort was very homogenous with similar age, fracture type, sex and surgical procedure in a single tertiary orthopedic center. Demographically our patients were older with an elevated risk of higher mortality and morbidity versus the average hip fracture population ( Table 1). All patients were included from the acute list and were operated upon during office hours.

Registry data of the Swedish hip fracture population demonstrates an average age of 82 and around 2/3 being female. ^2^ Thus, our patient cohort was seven years older and thereby probably frailer than the average hip fracture patient. A surrogate marker for frailty in this investigation was ASA grading (mean value 3) and the NHFS score (mean value 5), both slightly elevated *versus* the Swedish average. ^2^ Both scales assess comorbidity in various ways and are further used as prognostic scores for mortality risk in the perioperative and postoperative phase, like 30-day mortality. ^4^^,^^7^ Therefore, we claim to have succeeded in finding a patient population with high comorbidity, speculatively having a higher risk for intraoperative hypotension than the average hip fracture population.

## Conclusions

Our results confirm that spinal anaesthesia-induced hypotension is not solely a result of a fall in SVRI but mainly by systemic venodilation, reducing venous return to the heart and followed by a consequent fall in CO and MAP. The incidence of hypotension was low and only one third of the patients needed nor-epinephrine infusions all at modest doses, highest infusion rate was 0.12 μg/kg/min nor-epinephrine. Our results contradict the idea of spinal anaesthesia-induced hypotension as being solely a result of a fall in SVRI due to loss of sympathetic vascular tone.

## Authors contributions

FO: Planned and designed the study. Established intrathecal catheter and set up LidCO monitoring, collected and analyzed data. Wrote the first draft.

MHaS: Planned and designed the study and revised the manuscript

KD: Revised the manuscript

SER: Planned and designed the study and revised the manuscript

BN: Planned and designed the study and revised the manuscript

## Data Availability

Open Science Framework: Fractional anesthesia lidco, https://doi.org/10.17605/OSF.IO/XAGBY. ^39^ This project contains the following underlying data:
- fractionspinallidcoFO.csv Data are available under the terms of the Creative Commons Zero “No rights reserved” data waiver (CC0 1.0 Public domain dedication). Open Science Framework: TREND checklist for “Fractional spinal anesthesia and systemic hemodynamics in frail elderly hip fracture patients”, https://doi.org/10.17605/OSF.IO/D98VG. ^40^ Data are available under the terms of the Creative Commons Zero “No rights reserved” data waiver (CC0 1.0 Public domain dedication).
