# Supplementary material for: Fractional spinal anesthesia and systemic hemodynamics in frail elderly hip fracture patients
Source: F1000Res. 2023 Jul 31;12:210. Originally published 2023 Feb 24. [Version 3] doi: 10.12688/f1000research.130387.3 (PMC10366555; doi:10.12688/f1000research.130387.3)
Supplement: Peer review history

# Peer review: Reviewer response for version 3

**DOI:** 10.5256/f1000research.153803.r192515

**Published:** 2023-08-01

**Authors:** Luigi La Via

**Affiliations:** 1 Department of Anesthesia and Intensive Care, “Policlinico-San Marco” University Hospital, Catania, Italy

After the revision, I have no more comments to make.

Is the work clearly and accurately presented and does it cite the current literature?

Partly

If applicable, is the statistical analysis and its interpretation appropriate?

Yes

Are all the source data underlying the results available to ensure full reproducibility?

Yes

Is the study design appropriate and is the work technically sound?

Yes

Are the conclusions drawn adequately supported by the results?

Yes

Are sufficient details of methods and analysis provided to allow replication by others?

Yes

Reviewer Expertise:

Anesthesia; Critical Care

I confirm that I have read this submission and believe that I have an appropriate level of expertise to confirm that it is of an acceptable scientific standard.

# Peer review: Reviewer response for version 2

**DOI:** 10.5256/f1000research.147676.r184709

**Published:** 2023-07-24

**Authors:** Tony Kwun-Tung Ng

**Affiliations:** 1 Rehabilitation Sciences, The Hong Kong Polytechnic University, Hong Kong, Hong Kong; 2 Anaesthesia and Operating Theatre Services, Tuen Mun Hospital, Hong Kong, Hong Kong; 3 Anaesthesia and Intensive Care, The Chinese University of Hong Kong, Hong Kong, Hong Kong; 4 Anaesthesiology, The University of Hong Kong, Hong Kong, Hong Kong; 5 Frankston Pain Management, Melbourne, Victoria, Australia

Thank you for giving me an opportunity to review this article. I am personally a big fan of CSA in fracture hip patients although my regime is a bit different from your study regime.

 Here are my comments:

1. Did you measure the preoperative hydration status which is a crucial factor to affect the hemodynamic response after CSA?
2. What is the fluid management in conjunction of this technique?
3. Can you share with us the incidence of urinary retention after a total of 30mcg intrathecal fentanyl? I understand it is used to improve the quality of the CSA despite some retained motor function. Yet, it is a rather big dose to me.
4. Methodology wise, is there any reason to use 2 intrathecal boluses instead of a bolus followed by infusion? Infusion can supposedly provide an even more stable hemodynamic. 
5. Is there any evaluation of the timing to commence norepinephrine infusions for those 5 patients?
6. Concerning the FIC and Femoral nerve block, it is written down as 20-40ml diluted ropivacaine. Can you clarify which LA volume is used for which block? 40ml ropivacaine for femoral nerve block doesn't make sense to me in the presence of ultrasound guidance. 

Is the work clearly and accurately presented and does it cite the current literature?

Yes

If applicable, is the statistical analysis and its interpretation appropriate?

I cannot comment. A qualified statistician is required.

Are all the source data underlying the results available to ensure full reproducibility?

Yes

Is the study design appropriate and is the work technically sound?

Partly

Are the conclusions drawn adequately supported by the results?

Partly

Are sufficient details of methods and analysis provided to allow replication by others?

Yes

Reviewer Expertise:

Regional anaesthesia in hip surgery. Neurolysis in inoperable fracture hip. Use of opioid in pain management. Peripheral joint denervation.

I confirm that I have read this submission and believe that I have an appropriate level of expertise to confirm that it is of an acceptable scientific standard, however I have significant reservations, as outlined above.

# Response

**Published:** 2023-07-27

**Authors:** Fredrik Olsen

**Affiliations:** University of Gothenburg, Sweden

Thank you for taking the time and effort to review our work. 

 I have attempted to answer your questions point for point below. We see the shortcomings in not having a robust fluid protocol in place, a major limitation was conducting the study without disrupting the logistics of the department as a whole and some concessions needed to be made to the protocol.

 1. We did not quantify preoperative fluid status prior to inclusion.

 2. Fluid management prior to surgery is managed initially by the ambulance service/emergency department and then by the geriatric department. Patients were assessed by the study team on an individual basis with regards to time from hospital admission, assessed delay from fracture to hospital arrival and delivered fluids prior to arrival at the operating department. 

 3. No data on postoperative urinary retention was collected. Patients followed the ordinary postoperative routine after surgery. Through informal follow-up no reports of retention needing intervention was brought to our attention. This does not imply that it could have been an issue though.

 4. Regarding the use of bolus instead of continuous infusion; we attempted to mimic the use of low dose single shot spinal anaesthesia without our measurements being obscured by ongoing surgery. A spinal catheter enabled us to do that with out the need of administering a second single shot spinal for surgical anaesthesia. 

 5. Regarding timing of norepinephrine start; Those patients requiring NE infusion received in conjunction with the first dose. The second dose did not seem to trigger the need for a higher dose or the start of NE in patients not requiring it prior.

 6. The higher volume of LA volume (40ml) was used with a landmark based FIC block when ultrasound guidance was not available. With ultrasound guidance, 20ml was used for a targeted femoral block.

# Peer review: Reviewer response for version 2

**DOI:** 10.5256/f1000research.147676.r184705

**Published:** 2023-07-19

**Authors:** Michael Irwin

**Affiliations:** 1 The University of Hong Kong, Hong Kong, Hong Kong

An interesting and well conducted study. I think it is fairly well known that SA induced hypotension is multifactorial and that venodilation is an important factor ^1^. I would, therefore, describe this as confirmatory.

 Ref 12 is over 10 years old. Using LiDCO is a strength as is the use of plain bupivacaine (hyperbaric solutions should not be used in these frail elderly hip fracture cases in my opinion). The trail design was done to actually minimise the risk of hypotension and the results show that it will occur even with a meticulous technique (not CSA per se but use of a modest dose of plain local anaesthetic - cited refs 25 and 26 were not using plain bupivacaine).

 I suggest the conclusion should be " *Our results confirm that spinal anaesthesia-induced hypotension is not solely a result of a fall in SVRI but mainly by systemic venodilation, reducing venous return to the heart and followed by a consequent fall in CO and MAP. Using isobaric bupivacaine and CSA, the incidence of hypotension was low and only one third of the patients needed nor-epinephrine infusions all at modest doses"*.

 Minor: line 1 ' *Anaesthesiologic'* is not an accepted term. ' *A major challenge in anaesthesiology'* is better.

 A follow up study with single shot isobaric bupivacaine / fentanyl would be interesting as it is a simpler and quicker technique than CSA

Is the work clearly and accurately presented and does it cite the current literature?

Partly

If applicable, is the statistical analysis and its interpretation appropriate?

Yes

Are all the source data underlying the results available to ensure full reproducibility?

Yes

Is the study design appropriate and is the work technically sound?

Yes

Are the conclusions drawn adequately supported by the results?

Partly

Are sufficient details of methods and analysis provided to allow replication by others?

Yes

Reviewer Expertise:

Perioperative medicine; TIVA

I confirm that I have read this submission and believe that I have an appropriate level of expertise to confirm that it is of an acceptable scientific standard, however I have significant reservations, as outlined above.

## References

[1] : Control of Spinal Anesthesia-Induced Hypotension in Adults. *Local Reg Anesth* .2020;13: 10.2147/LRA.S240753 39-46 10.2147/LRA.S240753 32581577PMC7276328

# Response

**Published:** 2023-07-27

**Authors:** Fredrik Olsen

**Affiliations:** University of Gothenburg, Sweden

Thank you for taking the time to read and review our work.

 Your comments are to the point and relevant to the study. I have implemented parts of the text you suggested in the conclusion section and rewritten the line that contained the phrase "Anaesthesiologic" a per your recommendation.

# Peer review: Reviewer response for version 2

**DOI:** 10.5256/f1000research.147676.r171947

**Published:** 2023-05-05

**Authors:** Luigi La Via

**Affiliations:** 1 Department of Anesthesia and Intensive Care, “Policlinico-San Marco” University Hospital, Catania, Italy

I have no more comments to add.

Is the work clearly and accurately presented and does it cite the current literature?

Partly

If applicable, is the statistical analysis and its interpretation appropriate?

Yes

Are all the source data underlying the results available to ensure full reproducibility?

Yes

Is the study design appropriate and is the work technically sound?

Yes

Are the conclusions drawn adequately supported by the results?

Yes

Are sufficient details of methods and analysis provided to allow replication by others?

Yes

Reviewer Expertise:

Anesthesia; Critical Care

I confirm that I have read this submission and believe that I have an appropriate level of expertise to confirm that it is of an acceptable scientific standard.

# Peer review: Reviewer response for version 1

**DOI:** 10.5256/f1000research.143142.r164674

**Published:** 2023-04-11

**Authors:** Luigi La Via

**Affiliations:** 1 Department of Anesthesia and Intensive Care, “Policlinico-San Marco” University Hospital, Catania, Italy

I read with great interest the manuscript by Olsen *et al*. on the effect of fractional spinal anesthesia on systemic hemodynamics in frail elderly hip fracture patients. The authors investigated the changes in haemodynamic variables caused by two doses of intrathecal bupivacaine 2.25 mg and fentanyl 15 µg in 15 patients aged over 65. The paper is sound and well written. However, there are some minor issues to be addressed.

**Introduction**

- When you state "where many centers give general anaesthesia, while particularly in northern Europe, neuraxial anaesthesia is the preferred technique" you should add an appropriate reference (for example, Neuman *et al. *(2021) ^1^).
- Spinal anesthesia is also the preferred technique for other kind of surgeries ^2^^,^^3^. Please briefly discuss and add these 2 references. 

**Results**

- Please explain why "two patients were excluded due to logistical issues".

**Discussion**

- Please discuss the results of the recently published meta-analysis on the investigated topic ^4^.

**Conclusion**

- Considering the limitations of this study, I would state "The initial blood pressure decline after fractional spinal anaesthesia MIGHT BE caused mainly by systemic venodilation".

Is the work clearly and accurately presented and does it cite the current literature?

Partly

If applicable, is the statistical analysis and its interpretation appropriate?

Yes

Are all the source data underlying the results available to ensure full reproducibility?

Yes

Is the study design appropriate and is the work technically sound?

Yes

Are the conclusions drawn adequately supported by the results?

Yes

Are sufficient details of methods and analysis provided to allow replication by others?

Yes

Reviewer Expertise:

Anesthesia; Critical Care

I confirm that I have read this submission and believe that I have an appropriate level of expertise to confirm that it is of an acceptable scientific standard, however I have significant reservations, as outlined above.

## References

[1] : Spinal Anesthesia or General Anesthesia for Hip Surgery in Older Adults. *N Engl J Med* .2021;385(22) : 10.1056/NEJMoa2113514 2025-2035 10.1056/NEJMoa2113514 34623788

[2] : Spinal Anesthesia Provides Longer Administration Time for Postoperative Intravesical Chemotherapy after TUR-B Operation. *Urol Int* .2022;106(8) : 10.1159/000517790 768-774 10.1159/000517790 34333492

[3] : α-2 agonists vs. fentanyl as adjuvants for spinal anesthesia in elective cesarean section: a meta-analysis. *Minerva Anestesiol* .2022; 10.23736/S0375-9393.22.16969-5 10.23736/S0375-9393.22.16969-5 36448990

[4] : Spinal anesthesia and hypotensive events in hip fracture surgical repair in elderly patients: a meta-analysis. *Journal of Anesthesia, Analgesia and Critical Care* .2022;2(1) : 10.1186/s44158-022-00047-6 10.1186/s44158-022-00047-6 37386657PMC10245414

# Response

**Published:** 2023-04-30

**Authors:** Fredrik Olsen

**Affiliations:** University of Gothenburg, Sweden

**Thank you for taking the time and effort to review the paper, your comments are addressed individually in the following text and are reflected in the revisions of the updated draft.**

**Introduction**

- When you state "where many centers give general anaesthesia, while particularly in northern Europe, neuraxial anaesthesia is the preferred technique" you should add an appropriate reference (for example, Neuman *et al. *(2021) ^1^).
*Reference added.*
- Spinal anesthesia is also the preferred technique for other kind of surgeries ^2^^,^^3^. Please briefly discuss and add these 2 references.
*These two references seem only peripherally relevant to this study, and for the sake of brevity will not be included*

**Results**

- Please explain why "two patients were excluded due to logistical issues". 
*All participants had a preliminary time scheduled for their surgery and we performed the test protocol prior to this. If a slot in the program opened up the protocol stated that surgery would not be delayed to finish the study protocol. This was to keep the departments program flowing as smoothly as possible and to minimise potential harm to our patients.*

**Discussion**

- Please discuss the results of the recently published meta-analysis on the investigated topic ^4^. 
*The reference #31 has been changed to Messina 2022, which I suspect was the reference it was supposed to be all along. Nice observation from the reviewer!*

**Conclusion**

- Considering the limitations of this study, I would state "The initial blood pressure decline after fractional spinal anaesthesia MIGHT BE caused mainly by systemic venodilation".
*Edited in the revised draft to reflect reviewers comments*
